# Impediments to localization agenda: humanitarian space in the Rohingya response in Bangladesh

**DOI:** 10.1186/s41018-022-00122-1

**Published:** 2022-07-16

**Authors:** Abdul Kadir Khan, Tiina Kontinen

**Affiliations:** Department of Social Sciences and Philosophy, University of Jyväskylä, P.O.Box 35, 40014 Jyväskylä, Finland

**Keywords:** Localization, Humanitarian action, Humanitarian space, Civic space, Rohingya response

## Abstract

The article spotlights the impediments of the localization agenda in the Rohingya response in Bangladesh through the notion of humanitarian space. The Rohingyas rely entirely on material aid and humanitarian services in the camps, mainly stemming from international actors committed to the localization agenda, which, however, has not been effectively implemented. Drawing on the definition of humanitarian space as an arena, we investigate the main negotiations within humanitarian space in the Rohingya response and how they impede the realization of the localization agenda. We conducted secondary data analysis on reports published by organizations involved and validated the findings with ten telephone interviews with organizations in Bangladesh. We identified three main negotiations. First, negotiation on the nature of the partnership between local and international humanitarian actors, including the debates on the definition of “local”; second, negotiation concerning the characteristics and appreciation of local capacity; and third, the negotiation related to constraints within the operating environment for humanitarian actors in Cox’s Bazar, Bangladesh. We conclude that the lens of humanitarian space is necessary for further understanding the dynamics impeding localization in the Rohingya response in particular and humanitarian action in general.

## Introduction

Localization is the core agenda of current reform efforts in the humanitarian sector (Roepstorff 2020a). In the process of making the humanitarian response more effective, efficient, and emancipatory, during the World Humanitarian Summit (WHS) in 2016, a consensus was reached in the Grand Bargain^1^ for increased support and funding to local actors^2^ for strengthening leadership (Khaled 2021; Slim 2021a). In the Grand Bargain 2.0^3^ articulated in 2021, the overall objective remains relatively the same but prioritizes two areas. First, provide greater support for leadership, delivery, and capacity of local responders and the participation of affected communities in addressing humanitarian needs. Second, emphasize a critical mass of flexible support and “quality funding” for an effective and efficient response that ensures visibility and accountability. The conceptualization and realization of the localization agenda have been critically discussed in recent literature in both the development and humanitarian fields. For instance, Slim (2021a) argues that as opposed to merely being viewed as a means to increase effectiveness, localization should be understood as making humanitarian citizenship of the affected populations that requires self-determination and political justice by the international humanitarian leaders (ibid.). Pincock et al. (2021) show the discrepancy between the international policy rhetoric and the reality of localization regarding the inclusion of refugees. Roepstorff (2020a) problematizes the discourse of "local" as a binary opposition to international that fails to capture the complex dynamics of intervention processes. Hence, a critical localism framework has been suggested for analyzing the process by which the local is constructed (Lambek 2011; Mac Ginty 2015; Paffenholz 2015; Roepstorff 2020a). Additionally, Roepstorff (2020b) urges paying attention to protecting the humanitarian space and defending the humanitarian civic space by all actors involved in the humanitarian sector.

In a similar vein, this article puts forward the notion of humanitarian space in exploring the impediments of localization in the Rohingya response in Bangladesh. In general, humanitarian space has been discussed in relation to humanitarian action (Collinson and Elhawary 2012) and defined as a space amidst the practical humanitarian action and the norms articulated in the traditional humanitarian principles (Sezgin and Dijkzeul 2015). Additionally, humanitarian space is used to determine constraints arising from national migration policies, national laws, and other pressures from the government (Roepstorff 2020b). Furthermore, the term highlights the discrepancies while negotiating and navigating the space of humanitarian action between the local and international actors. This article draws on the literature which considers the humanitarian space as an “arena,” where continuous negotiations within everyday practices between different actors such as donors, UN agencies, international NGOs (INGOs), local NGOs (LNGOs), and a multitude of recipients of humanitarian aid shape the humanitarian action (Hilhorst and Jansen 2010), and the humanitarian space itself. Hence, we argue that it is crucial to explore how these negotiations within the humanitarian space restrict or impede the realization of the localization agenda.

The Rohingya response is an example of a humanitarian arena where different understandings of localization have been negotiated (Roepstorff 2021). Hundreds of thousands of Rohingya refugees fled to Bangladesh after the mass atrocities in Myanmar following the ARSA attack in 2017. Today, Rohingyas rely entirely on material aid and humanitarian services (CPJ and X-BORDER 2021). However, up to now, the localization agenda in the Rohingya response has failed to materialize in accordance with its commitments (Khan 2019; Van Brabant and Patel 2018b; Van Brabant et al. 2021). Although local actors were the “first responders” in the Rohingya crisis (Lewis 2019), today, they are dwarfed by the dozens of international aid agencies who dominate the donor funding and the emergency response^4^. Subsequently, Bangladeshi NGOs and civil society organization (CSO) leaders have been engaged in the campaign for localization, urging INGOs and UN agencies to recognize the local partners as a strategic partners and regard the “partnership with dignity” (Roepstorff 2021).

In light of the “as local as possible, as global as necessary*”* commitment to localization agenda and the extant analysis of the challenges in its realization, this article examines how the main negotiations are taking place and, simultaneously, shaping the humanitarian space that impedes the implementation of localization in the Rohingya response in Bangladesh. Based on the secondary analysis of qualitative data (Heaton 2008), building on the analysis of the so-called gray literature (Mahood et al. 2014), selected reports (*n* = 30) from organizations involved in localization in the Rohingya response in Bangladesh and ten supplementary interviews with representatives of Bangladeshi NGOs, we identify three main negotiations relevant for organizations. In what follows, we first articulate our conceptualization of humanitarian space in general and the situation regarding Rohingya in particular. Next, we proceed to describe the methods and materials used, after which we describe our findings of the three relevant negotiations shaping the humanitarian space relating to partnerships, capacities, and operating environments. In conclusion, we argue that amalgamating multiple and simultaneous negotiations shaping humanitarian space from the perspective of organizations provides a deeper understanding of the challenges of realizing the localization agenda in humanitarian action.

## Localization of humanitarian action: unpacking humanitarian space

Humanitarian action is perpetuated with the traditional humanitarian provision of immediate relief of human suffering in crisis (Dijkzeul and Sandvik 2019). Traditionally, humanitarian aid has been dominated by the UN agencies and INGOs, and centered around the classical Dunantist paradigm, which is based upon the ethics of humanitarian principles. However, in recent years, a resilience paradigm has been paralleled, promoting new humanitarianism which emphasizes the strengthening of local response capabilities (Hilhorst 2018). Thereby, discussions concerning humanitarian space have relinquished from being defined in accordance to international humanitarian law (Wagner 2005), and instead humanitarian space is being understood as an arena where diverse actors shape the everyday realities of humanitarian action (Hilhorst 2002; Hilhorst and Schmiemann 2002; Hilhorst and Jansen 2010; Hilhorst 2018). According to the localization agenda, local actors are key components in the orchestration of local responses. It is thus imperative to unpack the curtailment of their space for action in the humanitarian context (Roepstorff 2020b). In this section, we first review the discussion concerning localization and then articulate a nuanced understanding of humanitarian space in the Rohingya response.

### Reforming the humanitarian sector: more support and funding for local actors

At the World Humanitarian Summit (2016), a number of humanitarian organizations, donor countries, and aid agencies subsequently signed the agreement called “The Grand Bargain.” The second workstream of the deal, “more support and funding tools for local and national responders,” became known as “localization.” Some have argued it to be a remarkable failure (Slim 2021a) due to the absence of a definitive and widely accepted definition of localization (Van Brabant and Patel 2017). An OECD document of commitments to action defines *localization* as “a process of recognizing, respecting and strengthening the leadership by local authorities and the capacity of local civil society in humanitarian action, in order to address the needs of affected populations and to prepare national actors for future humanitarian responses” (OECD 2017). The desired shift raises questions about how international and local boundaries are drawn, how the humanitarian system can be attuned to local priorities (Harris and Tuladhar 2019), and how local capacity can be built while leveraging funds from international donors (Kraft and Smith 2019; Pincock et al. 2021). For the donors, localizing aid is more extensive than merely allocating more funds to local humanitarian responders. It also entails supporting local actors in changing how crises are managed, optimizing existing partnerships, and strengthening the voices of affected populations (Glennie, and Rabinowitz, a. G. 2013), by channeling donor funds directly to the local partners (Sundberg 2019).

Nevertheless the localization agenda has been criticized for placing the international humanitarian system at the center of the process (Jayawickrama and Rehman 2018). In practice, the process of localization remains anchored to a handful of UN agencies, donors, and NGOs from the West (Gómez 2021) rather than refocusing on local actors. Additionally, whilst affected populations are often recognized in the agreements made between the people, government, and institutions, the dominance of international aid agencies infringes the process, and the negotiation is diverted between government and international organizations rather than the affected people (Slim 2021a). Intrinsically, achieving localization requires profound reflection on how international norms and organizations interact with local ones, as well as how the culture and practices of international organizations interact with local practices (Pincock et al. 2021). Thus, localization requires in-depth knowledge of local contexts in their relationships.

Moreover, one of the initial infringements of the localization agenda is to define who the locals are (OECD 2017; Roepstorff 2020a; Roepstorff 2021). Usually, local refers to individuals and groups who are “the first responders,” almost self-evidently aware of the context, culture, and social fabric of affected communities (Schenkenberg 2016:10). However, the way the local is constructed is based on the problematic dichotomy between the local and international as a binary opposite (Paffenholz 2015; Roepstorff 2020a), leading to blind spots in the analysis of exclusionary humanitarian practices. In practical use, the label "local" refers to various actors proximate to national and local authorities, typically civil society organizations at the national and community level (Roepstorff 2021). Additionally, from an international perspective, the term “local actor” is often used to refer to national governments (Schenkenberg 2016). In order to reconceptualize the problematic dichotomy of “local,” Mac Ginty (2015) proposed “critical localism,” where the local is constructed beyond spatial notions of localism, not opposing international, and instead understood as an activity rather than a physical place (Mac Ginty 2015). Such contextualization, however, impinges on a wide variety of the ways in which the humanitarian actors in the field classify actors as being ‘local’.

Despite the challenges identified in the localization agenda, many actors have allegedly implemented it in diverse ways. The localization agenda also acts as a strategic form of legitimation, utilized by international and local actors to access funding (Roepstorff 2021). Many international civil society alliances and confederations create more national franchises of international NGO alliances, where many national CSOs change their identity and become part of the international brand (Van Brabant 2016). An international NGO can argue that if their country office is registered in the crisis-affected country, and is led or even fully staffed by nationals, it should be counted as a local organization. However, as Van Brabant (2016) suggested, one way to view such localization is as a business strategy of multinational (aid-based) corporations. In this regard, localization has become de facto a strategy of globalization that does not shift any power and may continue to undermine national or local capacities by establishing itself more forcefully in local markets (ibid.). Nevertheless, localization also takes place in a context characterized by institutional, legal, and political architecture, which shapes the relationships between international and local actors and the room for action for both. Therefore, we suggest the notion of humanitarian space provides an effective lens to make sense of the localization process in practice.

### Humanitarian and civic space in the humanitarian response

The concept of humanitarian space has been defined in various ways. Predominantly, in the context of conflict, humanitarian space is a way to measure humanitarian conditions that concern the international humanitarian law (Wagner 2005). Thus, it can be highlighted as a complex political, military, and legal arena where humanitarian action takes place (Collinson and Elhawary 2012). In its most fundamental sense, humanitarian space is an environment where humanitarians can work without hindrance and follow the humanitarian principles of humanity, impartiality, and neutrality (Collinson and Elhawary 2012; Hilhorst and Jansen 2010; Hilhorst 2018; Spearin 2001). It is not only a space for humanitarian agencies and their ability to access to people in need but also about the space of affected populations and their access to aid (Abild 2010), protection, and services provided and operated by the humanitarian agencies (Collinson and Elhawary 2012) impartially and independently.

For humanitarian organizations, the origin of the term is often attached to the former Médecins Sans Frontières (MSF) president Rony Brauman, who used the term *espace humanitaire* (Hubert and Brassard-Boudreau 2010) in 1990s. Based on this term, MSF calls for a “space for humanitarian” action in which aid agencies are “free to evaluate needs, free to monitor the delivery and issue of assistance, free to have a dialogue with the people” (Wagner 2005), but separate from any political aspect or influence (Hilhorst and Jansen 2010). Likewise, civic space in a humanitarian context is understood to be both a metaphorical and practical space where civil society actors work (Cunningham and Tibbett 2018). The humanitarian and civic spaces are connected, as the host country may introduce limitations to civic space while hosting a situation that needs humanitarian response. This may occur as a result of the host country feeling vulnerable to and/or threatened by external and internal political influence (ICVA 2019). Consequently, the politically restricted civic space can also manifest in demeaning the civil society response in emergencies (Roepstorff 2020b).

Ideally, the classic paradigm of humanitarianism is associated with the service delivery in temporary conflict situations, where the humanitarian principles are seen as socially negotiated (Sezgin and Dijkzeul 2015), symbolic and feasible within a physical space. Hence, many actors strategically use humanitarian principles to access the arena or discredit competitors (Hilhorst and Schmiemann 2002). However, adhering to the humanitarian principle is challenging for international and local actors (Trócaire 2018). For example, Collinson and Elhawary (2012) argue that adherence to humanitarian principles does not lie only with the humanitarian organizations but also with the political authorities and military forces (Collinson and Elhawary 2012). Therefore, in such contexts, the principles are continuously negotiated with other operation principles (e.g., partnership, participation, accountability) (Hilhorst and Jansen 2010). Hence, *humanitarian space* can be defined as an “arena” (Hilhorst and Jansen 2010). This perspective focuses on the everyday practices (Lewis 2019) of policy and implementation and highlights how different actors, including humanitarian and disaster-affected recipients of aid, negotiate with the universal normative values and shape the humanitarian action (Hilhorst 2018; Hilhorst and Jansen 2010). Thus, the principles become tangible in how the humanitarian responders interpret them and use them in everyday practice (Hilhorst 2002), in our example in the context of the Rohingya response.

### Localization in the context of the Rohingya response

Over a million Rohingyas live in congested, sprawling, fetid, and dire conditions, including the world’s largest and densest refugee settlement in Cox’s Bazar (Khan and Stensrud 2020; Lough et al. 2021). The Rohingyas are a *de jure* stateless group as their citizenship rights in Myanmar are not recognized by law (Sengupta 2021). Regardless of the recognition of Rohingyas as “refugees” by the UN systems (JRP 2018), they have not been registered as refugees or asylum seekers in Bangladesh. The authority continuously rejects their refugee rights and subjects Rohingya to various curtailments constraining the bounds of their everyday lives (Lough et al. 2021).

Due to apartheid in central Rakhine in 2017, Rohingyas fled to Bangladesh *en masse*. As a result, a level 3 emergency of international response was being called in Bangladesh (Bowden 2018), and Cox’s Bazar became the site of a large-scale humanitarian response (Roepstorff 2021). In the immediate arrival of Rohingya migrants, several charitable and informal voluntary efforts from a variety of individuals and groups responded in the form of “citizen aid,” “grassroots humanitarianism,” and “person to person helping,” also deployed as small-scale “everyday humanitarianism” (Lewis 2019; Richey 2018). However, within a few weeks, the humanitarian arena was transferred into a more tightly governed refugee space when a large number of international actors arrived in Cox’s Bazar and took control in collaboration with the government, army, and the UN agencies (Lewis 2019). Afterward, LNGOs were dwarfed by the massive “influx” of INGOs, and therefore, LNGOs became organized to jointly tackle their rights and interests for making their voices heard. Consequently, being local became a legitimizing factor for being involved in implementing “localization” in the Rohingya response (Roepstorff 2021:7).

From the beginning of the crisis, the government implemented a need-based response as opposed to a rights-based approach, considering the Rohingyas as passive recipients of aid rather than rights-holders of the response (Lough et al. 2021). Today, the Rohingya response is led and coordinated by the government of Bangladesh (JRP 2019 & JRP 2021). The crisis is overseen by the prime minister’s office of Bangladesh with other government ministries coordinated through a National Task Force chaired by the Foreign Secretary. The Refugee Relief and Repatriation Commissioner's (RRRC), along with Deputy Commissioner’s (DC) Office, leads the Rohingya response at the district level in Cox’s Bazar (Lough et al. 2021). Moreover, a sector-based coordination mechanism facilitates the overall humanitarian response, which is called the “Inter Sector Coordination Group” (ISCG). It is accountable to a Strategic Executive Group (SEG), which consists of leaders of humanitarian organizations, donors, and national NGO representatives, co-chaired by the UN resident coordinator, International Organization for Migration (IOM) and United Nations High Commissioner for Refugees (UNHCR) (Van Brabant et al. 2021). The Bangladesh government, however, from the onset of the influx, has been craving for emergency repatriation of Rohingyas, and it follows a temporary policy and approach to the Rohingya response (Roepstorff 2021).

In Bangladesh, the Rohingya crisis is not new; before the massive influx in 2017, many waves of Rohingyas sought refuges in Bangladesh, including in 1978, in the early 1990s, then in 2007, 2012, and 2016. However, in order to avoid providing the refugee status, and thus also the subsequent rights attached to the status (Lewis 2019), Rohingyas who fled after the 2017 influx are officially being called “forcibly displaced Myanmar nationals” (FDMN) (Van Brabant et al. 2021). In the past, Rohingyas` presence posed a security dilemma in Bangladesh, considering the rising challenges of fighting against Islamic terrorism and political Islam (Wolf 2014). Moreover, due to the geographical proximity to Myanmar, Cox’s Bazar is a red zone for narcotics, smuggling, and drug trafficking (Sengupta 2021). Based on these issues, the Bangladeshi authorities have framed the Rohingya as a security threat. Hence, the securitized approach of Bangladesh toward Rohingya has been affecting the humanitarian space (Sullivan 2021). The government has imposed many restrictions, including suspending NGO activities in the camps, and restricting visa approvals and general access of humanitarian workers. In addition, the unregistered or undocumented people living in the camps have no refugee rights in Bangladesh, and the government treats them as illegal immigrants under the Foreigners Act 1946 (ADSP 2020). Therefore, by denying Rohingyas the legal right to work, the Rohingya populations continue to rely solely on humanitarian aid.

Additionally, the NGO Affairs Bureau (NGOAB) of Bangladesh issued an order prohibiting cash-based programs (CPJ and X-BORDER 2021) in the camps and prioritizing the hiring of Bangladeshi nationals in the Rohingya refugee response (Sengupta 2021). The authorities fear that if Rohingyas have a “good life” in the camp, they will be less likely to leave the camps voluntarily (Khan and Stensrud 2020). Therefore, such attitudes have been manifested in the shrinkage of the humanitarian space in Cox’s Bazar (Lough et al. 2021). Although the Myanmar government has undertaken comprehensive bilateral and international diplomatic efforts in the wake of massive displacement in Bangladesh in 2017, a congenial environment for the dignified returns of Rohingyas in Myanmar has not been created. As a result, the Rohingya crisis in Bangladesh has become a protracted one, demanding continuous humanitarian action where international and local organizations work in a dynamic humanitarian space.

## Methods and data

To examine the main negotiations shaping the humanitarian space, we focus on the reported reflections and experiences of organizations involved in humanitarian response in the area. In addition, we scrutinize negotiations that, in one way or another, impede the localization process in the Rohingya response. To identify relevant negotiations, we used two kinds of research material: First, we analyzed a set of reports (*n* = 30) published after the onset of the Rohingya influx in Bangladesh in 2017, produced by both local and international organizations. Second, we conducted telephone interviews (*n* = 10) with representatives of the Bangladeshi civil society organizations involved in the humanitarian response to validate the results of our review of secondary data.

In this regard, we used a systematic humanitarian database search and snowballing to identify the existing, so-called gray literature (Mahood et al. 2014) that included evaluation reports, annual reports, working papers, and policy briefs. We conducted a systematic humanitarian database search in the “Overseas Development Initiatives” (ODI), “Reliefweb,” and “humanitarian response” by inputting the four keywords “humanitarian space,” “localization,” “humanitarian action,” and “Rohingya response” including the search term AND/OR owing to the publication after 2017 onwards. After screening the initial lists of literature and its bibliography, we diverted our search on snowballing for both local and international organizations’ websites. From this process, we identified those discussing the localization agenda and the space of humanitarian Rohingya response after the onset in 2017. Finally, we found 30 documents from different secondary sources (see Table 1) suitable and informative for our research aim. In addition, to verify and complement the secondary data analysis, we conducted ten telephone interviews with the leaders of local civil society organizations associated with the publication of local reports. The semi-structured interviews discussed their experiences on the themes concerning humanitarian space and localization identified in the secondary data analysis.

**Table 1:** Selected documents for analyzing localization discourse based on Rohingya response after 2017

Documents source	Authors/source
Global Mentoring Initiative (GMI)	We still need to talk — ToGETHER (Van Brabant et al. 2021); Debating Grand Bargain in Bangladesh (Van Brabant and Patel 2018a); Going the extra mile (Patel 2017).
CCNF/COAST Bangladesh	Business as usual or breaking the status quo (COAST 2016); a social review on Rohingya crisis long-term action plan (COAST 2020b); long-term action plan is needed based on joint risk assessment (COAST 2020a); crisis within the crisis (COAST 2018a); localization approach for Rohingya response (COAST 2018c)); fast responders are kept far (COAST 2018b).
Refugees international	A voice in their future (Sullivan 2020); fading humanitarianism (Sullivan 2021); aid restriction endangering Rohingya ahead of Monsoon in Bangladesh (Sullivan 2018)
Act for peace	An agenda for dignified Rohingya response (Sengupta 2021)
Asia Displacement Solutions platform (ADSP)	Rohingya in South East Asia Part 1 & 2 (ADSP 2020)
Overseas Development Institute (ODI)/Humanitarian Policy Group (HPG)	Participation and inclusion in the Rohingya response (Lough et al. 2021); the Rohingya response in Bangladesh and the global compact on refugees (Hargrave et al. 2020); capacity and complementarity in the Rohingya response in Bangladesh (Wake and Bryant 2018); the Rohingya crisis: making the transition from emergency to longer term development (Wake and Yu 2018); Rohingya refugees in Bangladesh: the humanitarian response (HPN 2018); rethinking capacity and complementarity for a more local humanitarian action (Barbelet 2019)
Center for peace and justice (CPJ)/BRAC University	Navigating at the margins (CPJ et al. 2020), Localization Roadmap (CPJ 2020/draft); views of Rohingya refugees (CPJ and X-BORDER 2021)
International crisis group	A sustainable policy for Rohingya refugees (ICG 2019)
Strategic executive group (SEG)/Joint response plan for Rohingya humanitarian crisis	(JRP 2018); (JRP 2019); (JRP 2020); (JRP 2021)
National Alliance of Humanitarian Actors Bangladesh (NAHAB)	State of humanitarian actions in Bangladesh (NAHAB 2020)
=10	=30

Drawing on ideas from thematic analysis (Braun and Clarke 2006; Braun and Clarke 2021), we first thoroughly reviewed the material on the final literature list to get an overall idea of their contents, using the MS Word file for screening and annotation. Then, we manually coded the contents systematically, focusing on the diverse elements of the Rohingya response. Finally, we combined the detailed contents into three main broader themes, which illustrate three main negotiations shaping the humanitarian space and impeding the realization of localization. Identified negotiations were as follows: first, negotiation on the nature of the partnership between local and international civil society actors, including the debates on the definition of “local”; second, negotiation concerning the characteristics and appreciation of the local capacity; and third, the negotiations related to constraints within the operating environment for humanitarian actors in Cox’s Bazar, Bangladesh. When reporting our findings, we will reference the analyzed studies and reports according to their individual or institutional authors, as listed in Table 1. The reports are also listed under the list of references.

## Results and discussion

In this section, we will present the findings concerning three main negotiations identified based on the analysis of the documents, verified and enriched in the interviews. We first discuss how the implication of the localization agenda is circumscribed by unequal partnerships between local and international organizations, and how further ambiguity exists in defining local in those partnerships. Second, we discuss the disparities of local capacities as negotiated within localization, related to local organizations’ adherence to humanitarian principles and their overall capacity amidst the Rohingya response. Third, we discuss how the localization process takes place in an operating environment where both international and local humanitarian actors need to negotiate their humanitarian principles and action amidst restrictive policies and burdensome bureaucracy of Bangladesh. In addition, how the access to, and role of the affected Rohingya people is restricted in this setting is also discussed.

### Unequitable partnership: negotiating the roles and definitions of “local”

The Rohingya response is the first major intervention since the humanitarian sector committed to work “as local as possible, as international as necessary” (Barbelet 2018). A series of publications from a local Bangladeshi organization, “COAST trust,” proclaimed that most of the INGOs and UN agencies consider local NGOs in the Rohingya response as mere implementing partners in contrast to seeing them as decision-making partners in their operation in Cox’s Bazar (COAST 2016; COAST 2018a; COAST 2018c; COAST 2020a). As a response, the representatives of the local civil society organization in Cox’s Bazar demanded a more localized response (Barbelet 2019) and equitable partnership grounded on the Principles of Partnership (2007)^5^.

In Bangladesh, the agenda of localization in the Rohingya response was adopted in December 2017, and the Grand Bargain localization workstream mission was raised in September 2018 (Van Brabant et al. 2021). However, even after four years of humanitarian operation since 2017, there is still no functioning space for the humanitarian actors. The gradual reduction of funding enhances the localization by default but not by design (Van Brabant et al. 2021). A study of the first 100 days of the Rohingya response (Shevach et al. 2018) reveals that national organizations received only 4% of funding (Harris and Tuladhar 2019) where the majority of 69% was allocated to the three UN agencies, followed by 20% share to the INGOs, 7% goes to IRRC (Khan 2019). The channeling was far below the commitment of sharing the direct funding in the Grand Bargain and the pledge made at Charter for change (c4c)^6^.

Even if the international NGOs and UN agencies are committed to increasing funding to the local and national NGOs in the humanitarian response, they are not fulfilling their commitments to promoting equitable partnership. For example, in the Grand Bargain workstream mission to Bangladesh, there is no Bangladeshi co-leadership (Van Brabant et al. 2021). In Lough et al. (2021), one of the leaders of the LNGOs decried, *“We are an implementing partner, the design is the responsibility of the UN agency or the sector group, and we are the implementation partner. As an implementing partner in an emergency, there is limited opportunity to ensure participation”* (Lough et al. 2021:35). Furthermore, although most INGO staff in Cox’s Bazar are Bangladeshi nationals (JRP 2018), an uneven power dynamic is visible in two-tier hiring practices and the different pay scales (Barbelet 2018).

Intriguingly, the Bangladesh government requires an essential engagement between local and international actors, giving more power and legitimacy to the local actors as international actors need to partner with a national actor to gain access to the refugee camps (Barbelet 2018). Accordingly, there is competition among the local civil society organizations to become an implementing partners with the INGOs. Local NGOs want their candidacy to be enlisted in INGOs’ organizational portfolio, which then can seal strong candidateship for other projects. Henceforth, most local NGOs tend to speak about the equitable partnership with the donors in an apprehensive manner, despite their experiences with inequalities to avoid cutting off future funding for their following operating projects. In this view, one of the interviewees describes the situation:

They (INGOs) are talking about the spirit of partnership to promote localization. We do not have any decision-making power. We are mostly relying on foreign funds to implement their project. They have their foreign staffs who are highly paid, whereas we are local, and we know the culture, language, and the context. However, we got a low salary. We cannot raise our voice because we are competing with other local actors, and we are fearful if they deny our next project replacing to another local organization. (participant 6, local NGO, Rohingya response)

In practice, INGOs struggle to find and select suitable local partners, leading to rivalry between different LNGOs, which has been described as “competitive humanitarianism” (Roepstorff 2021; Stirrat 2006). Although a general spirit in localization might be strengthening the power, and influence to the local partners to work instead of the foreign experts in a host country (Sundberg 2019), the donors still need intermediaries to manage multiple partners. The localization mechanism should be based on complementarity and partnership irrespective of the size and power of the local NGOs and not primarily on sub-contracting, risk transfers, and exploitation of local actors. Unfortunately, many INGOs tend to directly implement their projects despite the envisioned complementarity. Thus they perversely sideline local expertise and knowledge.

Another potential challenge in the partnership approach is the vagueness of the term local. The question of who should be counted as “local” is a source of tension that further complicates the understanding of the concept of localization. The conceptualization of the local is not only a theoretical exercise but has important implications for humanitarian practice (Roepstorff 2020a). Local refers to national-level and community-based actors, but as the term “ultra-local” emerged (Barbelet 2018), the definition of the term ‘local’ became more perplexing and appealed further debates. Similarly, in Cox’s Bazar, Roepstorff (2021) elucidates the nuances between “local” and “real local organizations”; the “very local,” or the “really really local” organizations in the Rohingya response, in reference to the organizations which were small in size with limited capacity and resources and originated in Teknaf or Ukhiya (Roepstorff 2021:9). In Bangladeshi "local" aid discourse broadly refers to international, national, and local agencies that have long been in Bangladesh. Bangladesh has been home to many international NGOs since its independence in 1971, where many national NGOs have engaged in the development sector for over 20 years (Sengupta 2021). Even if many of them lacked experience in refugee response (Barbelet 2019), they are now working with the Rohingya crisis (Patel 2017). For example, Bangladesh Rural Advancement Committee (BRAC) originated in Bangladesh in 1971 and now operates in more than ten countries. Despite its international presence, BRAC is considered a national Bangladeshi NGO within the humanitarian coordination of the Rohingya context due to its origins and policymaking influence in Bangladesh.

The IASC^7^ Humanitarian Financing Task Team (HFTT) set up the “localization marker working group” in July 2016 after the consultation process and recommendations from the consensus reached by Grand Bargain signatories. In 2018, it agreed to develop and apply a “localization marker” 8 for measuring direct and indirect funding to local and national actors. The proposed definitions can be characterized to be of two types. First, *local and national non-state actors* refer to relief organizations with headquarters that operate in their own aid recipient country, including the National societies of Red Cross and Red Crescent, which are not affiliated with an INGO. Second, *national and sub-national state actors* refer to the state authorities of the affected aid recipient country engaged in relief at the local or national level (e.g., RRRC in Bangladesh). Additionally, other types of organizations emerge in the discussion, for example, *internationally affiliated organizations*, which are affiliated to an international organization through inter-linked financing, contracting, governance, and decision-making systems. However, this category does not include local and national organizations that are part of networks, confederations, or alliances (OECD 2017; Van Brabant and Patel 2017). For instance, INGOs in Bangladesh can easily re-design their organizational structure and nationalize their country offices by a confederation structure. In this way INGOs legitimize and entitle them to receive direct funding agreed in Grand Bargain. One of the local practitioners explains:

International organizations can easily nationalize in Bangladesh with the confederated structure of their country office if they have 25 Bangladeshi board members in their country offices and are registered as per the guideline of NGO registration by NGOAB. INGOs can easily claim them as national NGOs and become eligible for direct funding committed in the Grand Bargain. However, it is not a good way of promoting localization. (Participant 3, Local actor)

Moreover, *southern-based NGOs* include those whose headquarters are not in an OECD^9^, and DAC^10^ member countries (Van Brabant and Patel 2017) and carry out operations outside the aid recipient country in which they are headquartered and are not affiliated with an international NGO (OECD 2017). In this way, the same organization can be classified as a national organization when carrying out operations within the country of origin. BRAC from Bangladesh could be an example of a southern-based organization, but it acts as a national agency when it responds in Bangladesh. Conversely, when it works in other foreign countries, it can be categorized as a regional or international organization (Van Brabant and Patel 2017. Additionally, as Bangladesh has prevented the acknowledgment of refugee-led response, Rohingyas are not considered local actors in this context (Wake and Bryant 2018), and neither have the recognition as humanitarian citizenship (Slim 2021a). Nevertheless many Rohingya-led organizations, youths, and social advocacy groups working in the camps have limited space to work as local actors. Notwithstanding being the main affected population in the crisis, Rohingyas neither have any representation in the sector meetings nor have they been consulted by the humanitarian service providers (CPJ and X-BORDER 2021).

In conclusion, the review of the reports and interviews revealed that Bangladeshi CSOs, who are self-evidently part of the "local" category, have experienced inequalities in their partnerships with international actors but at the same time, are being forced to compete against each other for entering in such partnerships. However, the overall category of “local” is contested, and there is constant negotiation over who is included as a “legitimate local.” Moreover, while continuous negotiations are underway on shaping the notion of “local” in humanitarian space, which directly affects the means of actualizing localization, the most significant challenge is the exclusion of the affected population, Rohingya, from the space of humanitarian action.

### Discussing the local capacity: a unique asset or in need of capacity building

In the localization agenda, local capacity is considered one of the main assets in enhancing the complementarity and partnership. Therefore, one of the essential capacities of LNGOs is to consider their “local knowledge,” which should be prioritized in designing the Rohingya response. Concurrently, the capacity of LNGOs is constantly portrayed as in need of capacity building in order to realize their role in partnerships. Practically, rather than providing capacity building for LNGOs, INGOs tend to poach their best staff to work for them (Barbelet 2019; COAST 2018b). In the Rohingya response, many local staff with local knowledge shifted from local NGOs to international ones for better salaries and opportunities, further blurring the lines between local and international (Barbelet 2018). Regardless of considering the local offices of INGOs with the majority of local staff as “local capacity” (Wall and Hedlund 2016), the short-term hiring processes, however, undermine the capacity of local NGOs by preventing them from building on past project outcomes or maintaining trained staff in their ongoing programs (Wake and Bryant 2018).

Another aspect of capacity, stemming from a slightly different understanding, is the ability to adhere to certain general humanitarian norms and values such as independence and do no harm, principles such as core humanitarian standards, and approaches such as right-based and people-centered approaches should be grounded on comprising protection of space in the Rohingya response (Barbelet 2018). However, most local organizations are not purely humanitarian, adhering to the core humanitarian principles (Labbé and Daudin 2015; Roepstorff 2020b; Schenkenberg 2016; Wall and Hedlund 2016). In general, local and national actors find the application of several of the principles challenging, and it has been argued that there is no situation where humanitarian action is entirely principled (Hilhorst and Schmiemann 2002; Schenkenberg 2016). For the local actors, engaging with the religious, economic, and political affiliations regarding the root causes of conflict is natural. Likewise, Bangladeshi LNGOs are often highly politicized as many are under the patronage of different political parties and ministries. Thus, many local actors cannot segregate their activities from partisan politics, advocacy, or expressions of solidarity. In this regard, Cox’s Bazar can be perceived as a politically and socially conservative district and a hardworking area for CSOs seeking to promote progressive social rights (ADSP 2020).

In Bangladesh, a response from local faith-based organizations (Muslim charity groups) was significant at the beginning phase of the refugee exodus (Lewis 2019). However, later, Bangladeshi government suspended several faith-based NGOs and Muslim charity activities (Ansar and Khaled 2021; Lewis 2019), claiming they pose threats to state security considering the radicalization of Islamic terrorist groups in the camps. In the opposite view, Patel (2017) observed that,

Most national civil society organizations consider themselves more than just service deliverers as of the governance dynamics in their country. A connection to a political party is not an automatic indicator that the agency will not be willing or able to adhere to humanitarian principles. Political connections may be used to protect the integrity of the relief operation (Patel 2017:24).

It is challenging to consider the extent to which local and national actors adhere to humanitarian principles since the role of local actors is unobserved and, therefore, less accountable for the principles than the international actors that explicitly build their agendas on them (Fast and Sutton 2018). Furthermore, when the humanitarian space is understood as an arena where humanitarian action takes place in the “everyday realities” (Hilhorst and Jansen 2010; Lewis 2019), the humanitarian action is not predetermined primarily by humanitarian principles. Instead, it derives from how the service delivery conditions in crises are shaped in everyday practice (Sezgin and Dijkzeul 2015). Therefore, one can argue that principled humanitarian action in its traditional sense succumbs to the localization agenda if the local capacity and contextualized knowledge are prioritized.

In this regard, Wake and Bryant (2019) found few respondents from INGOs and UN agencies who criticized LNGOs’ resistance to assimilating into the formal humanitarian system for not adhering to its normative values and expectations (Wake and Bryant 2018). On the contrary, some argue that neutral humanitarianism is not ethically desirable as legally, morally, or ethically as one can take sides for good reasons and still be humanitarian (Slim 2021b). At the same time, it is noteworthy that the international community involved in the Rohingya response, whether UNHCR or IOM, never took a strong and principled stance to protect the Rohingya rights regardless of their status as “refugees” or as “forcibly displaced” people (Van Brabant et al. 2021). International NGOs and UN agencies acknowledged limitations in their capacity regarding mastery of local languages, being geographically proximate enough, and understanding the local communities, which guided them to rely on the capacity of local actors to build the entry points during emergencies (Wake and Bryant 2018). Thereby the capacity of local NGOs increased due to the complementarity and partnership with INGOs. However, one of the respondents in the interview postulates that

NGO Platform is a body of over 130 INGOs working in the humanitarian Rohingya response. Any organization for its membership needs to be onerous to the forum’s ethical values and obligations and follow the principles in its membership form. (Participant 9, local practitioner)

In this way, local capacity can be enhanced through the complementarity and partnership with the international organizations for dealing with humanitarian action in a principled manner. Even in the Grand Bargain, the partnership has been elucidated to reinforce the local capacities and not replace them. Lough et al. (2021), in this regard, ponder the risk grounded with the direct control of programming in the hostile operating environment with the government in the Rohingya context — as any handover of power to local NGOs would provoke a negative response for the government resulting in further challenges on the already tight humanitarian space (Lough et al. 2021).

Overall, the analysis of the reports and interviews revealed the multiple negotiations and dilemmas concerning the local capacity revolved around three main issues. First, international organizations’ appreciation of knowledge and capacities in local organizations but reluctance to build the capacity in support of complementarity instead attract the knowledgeable staff to be employed by INGOs. Second, there is a continuous negotiation around local organization` (im) possibility to adhere to the traditional humanitarian principles and thus, be firmly integrated with the humanitarian system while rigidly embedded in the country`s political, social, and religious fabric. Here, when defined based on humanitarian principles, the negotiation around the humanitarian space tends to work against the inclusion of local organizations through complementarity and partnerships.

### Hostile operating environment: negotiating localization in a shrinking humanitarian space

The third negotiation concerning humanitarian space and localization in Rohingya response that emerged in our analysis was the hostile operating environment imposed by the Bangladeshi government, both towards the Rohingya, humanitarian organizations, and civil society organizations at large. Undeniably, the government`s short-term strategy for Rohingya treatment is focusing on voluntary repatriation, and the government views the crisis with fear and uncertainty as- if the Rohingyas have a better life in the camps, they will never return to Myanmar. Since the only government strategy is repatriation, the humanitarian space is negotiated between the state and the international agencies while the humanitarian citizenship of the affected Rohingya population is ignored.

The exclusion of the Rohingya from negotiations in the humanitarian space is also actively advanced by the government in restricting both the local and international activities with the affected population. The sheer scale and concentration of the crisis in Bangladesh have motivated Bangladeshi authorities to limit both Rohingya rights and the humanitarian space (Lough et al. 2021). A report from the International crisis group (2019) states how the Bangladesh government`s restrictive policies affect the humanitarian response (ICG 2019). Bangladesh`s restrictive policies include- relocating thousands of Rohingyas to the remote fragile island in Bhasan char (ADSP 2020), forbidding cash-based aid^11^, denying the Rohingya refugees legal employment rights (CPJ and X-BORDER 2021), and allowing humanitarian access to the camps only between 9 a.m. and 5 p.m. (Hatdash 2021). Furthermore, claiming to enhance state security, Bangladesh built barbed wire fences around the camps (Lough et al. 2021; Sullivan 2021), aiming to control the perimeters of Rohingyas.

The Bangladeshi government also restricts and controls which INGOs are allowed to enter the country (Van Brabant et al. 2021). Additionally, the government has exercised several measures to restrict NGO activities in the camps. For example, in 2019, the parliamentary standing committee on the foreign ministry of Bangladesh banned 41 NGOs from working at Rohingya camps (Sengupta 2021). Besides, the humanitarian actors face direct and indirect threats to NGO operations, delayed project approvals, and increased surveillance and scrutiny of the camp projects and staff (ibid.). The Bangladesh Parliament passed the controversial foreign donations (voluntary activities) regulation act 2016 (Act no. 43)^12^, which regulates the work and activities of foreign-funded NGOs. Eventually, the global civil society alliance (CIVICUS)^13^ alleged that Bangladesh’s new foreign donations law is in breach of international norms and agreements, and it will have serious negative consequences for Bangladeshi civil society and prevent NGOs from undertaking their essential and legitimate work. Furthermore, the draft Volunteer Social Welfare Organizations (Registration and Control) Act 2019 has raised serious concerns about the civic space of NGOs delivering mandates independently. Also, the Digital Security Act 2018 is used against media and Civil Society groups to curtail their freedom of speech and expression (Sarkar 2020). In the Rohingya response, the foreign NGOs seeking to work in Bangladesh must go through the FD6 and FD7 forms registration process with the NGO Affairs Bureau of Bangladesh (NGOAB)^14^ to be allowed to operate in the camps (Wake and Bryant 2018). Any entity using foreign funding must fill out lengthy foreign donation forms, so-called FD6s for involvement with development projects and FD7s for emergency response projects (Sullivan 2018). Moreover, each application is labor-intensive and requires a detailed budget and material information, target beneficiaries, and geographical level, including the Union level budget and activities to be clarified. The approval, nevertheless, takes several weeks and is subject to changes by government authorities. One of the participants stated that:

At the end of March 2021, a massive fire broke out in Kutupalong Balukhali camp in Cox’s Bazar, where many Rohingya died, and around 12000 shelters were destroyed and damaged. Those Rohingyas became homeless immediately, but it took almost four months for the project approval to make a shelter for those homeless people. (Participant 4, local practitioner)

Notably, any project with FD7s cannot be approved for more than six months^15^ at a time. Exceptionally, the UN-funded JRP (joint response plan) can be operated for a maximum duration of twelve months. Furthermore, the current directives stipulate that any organization with any FD7 project must submit a separate project proposal for the FD6 project, where 25% to 30% of the total project fund of any FD7-funded project for Rohingya camp is allocated for host communities in Cox Bazar. This aligns with other refugee-hosting countries` policies according to which host communities should be included in humanitarian actions, for instance, in the official government strategy regarding the humanitarian needs of the South Sudanese refugees living in Uganda (Dijkzeul 2021). Nevertheless, upon completion of the project, the project leader must submit the closure report, along with several levels of bureaucratic approvals and permissions, including an FD (Foreign donation) audit, the recommendations from the Upazilla Nirbahi Officer (UNO), and the Deputy Commissioner (DC) offices in Cox’s Bazar, which many project leaders find time-consuming and complex. Localization in such negotiated humanitarian space should not be assessed by benchmarking the overall goal and pledge made in different localization agreements (e.g., Grand Bargain, C4C); instead, nuanced understanding is needed for the space of action.

These negotiations concerning humanitarian space vis-á-vis state authority resonate with the tendency to constrain civic space in Bangladesh and elsewhere (Roepstorff 2020b). The strategies related to registration and detailed control, limits for foreign funding, as well as harassment, echo those used in other countries where, according to CIVICUS (ibid.) monitor, civic space is shrinking. However, there are some particularities over the humanitarian space in the specific case of Rohingya where restrictions are not only geared towards organization but most fiercely towards the affected Rohingya population who are excluded in the negotiation for the humanitarian space.

## Conclusion

 This article aims to examine the main negotiations that take place in and simultaneously shape the humanitarian space as an arena in the context of Rohingya response in Bangladesh and potentially limit the realization of the localization agenda articulated in the Grand Bargain. Focusing on the organizations` point of view and analysis of their reports on the Rohingya response, we identified three negotiations shaping humanitarian space. First, negotiation concerning the nature of collaboration between local and international organizations, including the debates over the definition of “local.” Second, negotiation relates to local organization`s capacity, appreciation, building, and adherence to humanitarian principles. Third, negotiation focused on the operating environment enabled and constrained by the Bangladeshi government policies and bureaucracies.

Each negotiation is related to the realization of the localization agenda and presents particular impediments to its rigorous implementation. Our findings support Roepstorff`s (2020b) elucidation of how the misconceptions and divergent understanding of localization hampered joint efforts of both local and international actors in the Rohingya response (ibid.). Building collaboration relations between international and local organizations based on sub-contracting rather than complementarity, hierarchy rather than an equal partnership, and modifying international organizations to be “local” hinders the local agency within the humanitarian space. Further, while the localization agenda emphasizes local knowledge and complementarity of international and local actors` capacities, the international organizations absorb human capacity and diminish the local capacity due to their failure to adhere to traditional humanitarian principles. As a result, they hinder the realization of localization by limiting the capacity and excluding local organizations from the humanitarian space. Thus, based on our findings, we can agree that localization requires trust-building efforts (Roepstorff 2020b) and self-determination (Slim 2021a) for a dignified partnership. Moreover, localization in humanitarian aid is taking place in specific national contexts that shape the kinds of organizations and activities allowed. Therefore, the “localization” efforts of the humanitarian actors are easily limited and hampered by the restrictions introduced in the legal and bureaucratic context.

The article`s main contribution is to introduce and utilize the concept of humanitarian space as an arena of negotiations in discussing the localization agenda. Since there is no agreed definition for either the concept of localization or the humanitarian space, it is imperative to realize how the humanitarian action can be more effective and inclusive, where the entire humanitarian system needs to be turned on its head (Gingerich and Cohen 2015). Although, the systematic shortcoming of local turn is that it keeps the international humanitarian system at the center of the process. In the hierarchical process of the humanitarian system, most official humanitarian aid is financed by the West, and the money, as well as power, is transferred directly to the international agencies (Slim 2021a). With this process, the Western governments and a few INGOs largely dominate the global humanitarian policymaking and aid allocation in crisis.

Thus, a reform effort of “as local as possible and as international as necessary” commitment of WHS is rhetorically described as a partnership and capacity building of local actors, where the Western agencies legitimize their hierarchical position of power in the humanitarian system. In such a relationship of sub-contracting partnership, a few local actors have access to the space of action. In this regard, the implementation of localization builds upon the negotiations mainly between the government and international agencies, where local actors have a weaker possibility of the primacy for the negotiations in the humanitarian space for action. Additionally, due to the constrained settings by the host government, humanitarian space is shrinking in the Rohingya response, and vis-à-vis civic space is changing.

The main limitations of the article are related to mainly using secondary data to identify the main negotiations, partly dictated by the restrictions caused by the global COVID-19 pandemic. However, the studies with the secondary data we analyzed were produced by the organizations involved in the response and validated and enriched by local organizations in the interviews; we are content that our findings reflect the relevant negotiations, which pave the way for future empirical investigations.

More research will be needed on the negotiations in everyday practices where a variety of organizations seek a legitimate position of “local” organizations to benefit from the localization agenda. In addition, a nuanced analysis of the local responses outside the “humanitarian space” constructed by international agendas, humanitarian organizations, and the Bangladeshi government is needed, such as everyday humanitarianism (Richey 2018) by the local populations and the camp residents. Finally, it is imperative to examine how Rohingyas become humanitarian citizens (Slim 2021a) and thus, be included in the humanitarian space in alignment with the localization agenda.

## Data Availability

The secondary data generated or analyzed during the current study are publicly available. The interview data are restricted due to the protection of personal data of the recipients but are available from the corresponding author on reasonable request.

## Abbreviations

ARSA: Arakan Rohingya Salvation Army
BRAC: Bangladesh Rural Advancement Committee/Building Resources Across Communities
CCNF: Cox’s Bazar CSO-NGO Forum
C4C: Charter for Change
COAST: Coastal Association for Social Transformation Trust
CPJ: Center for Peace and Justice
CSO: Civil Society Organizations
DC: Deputy Commissioner
ICRC: International Committee of the Red Cross
IOM: International Organization for Migration
INGO: International non-governmental organization
ISCG: Inter Sector Coordination Group
JRP: Joint Response Plan
LNGO: Local non-governmental organization
MSF: Médecins Sans Frontières
NGOAB: NGO Affairs Bureau
OECD: Organization for Economic Co-operation and Development
RRRC: Refugee Relief and repatriation commissioner
SEG: Strategic Executive Group
UN: United Nations
UNHCR: United Nations High Commissioner for Refugees
UNO: Upazilla Nirbahi Officer
WHS: World Humanitarian Summit

## Footnotes

1 The Grand Bargain — a shared commitment to better serve people in need, available in http://agendaforhumanity.org/sites/default/files/resources/2018/Jan/Grand_Bargain_final_22_May_FINAL-2.pdf, accessed 26.08.2020

2 In this paper, we use the term local actors to refer to both locally originated and national non-governmental organizations (NGOs), civil society organizations (CSOs), and non-state actors.

3 The Grand Bargain 2.0, endorsed framework and annexes, June 2021, see https://interagencystandingcommittee.org/system/files/2021-07/%28EN%29%20Grand%20Bargain%202.0%20Framework.pdf, accessed 14.07.2021

4 Foreign aid is a hindrance to Rohingya repatriation, see https://www.dw.com/en/opinion-foreign-aid-is-a-hindrance-to-rohingya-repatriation/a-50157682, accessed 20.03.2020.

5 The Principles of Partnership (2007), available in https://www.unhcr.org/5735bd464.pdf, accessed 10.08.2020

6 The Charter for Change (C4c) is an initiative that aims to enable local and national actors to play a stronger role in humanitarian response by changing the way the humanitarian system works. Available at https://charter4change.org/. Accessed 10 Aug 2020

7 Inter-Agency Standing Committee facilitates the leadership role of United Nations Secretary Generali. It is the highest-level standing forum, see https://interagencystandingcommittee.org/ , accessed 01.01.2021

8 IASC Humanitarian Financing Task Team, Localization Marker Working Group, see https://interagencystandingcommittee.org/system/files/hftt_localisation_marker_definitions_paper_24_january_2018.pdf , accessed 15.07.2020

9 Organization for Economic Co-operation and Development is an intergovernmental economic organization with 38 member countries, see http://www.oecd.org/about/members-and-partners/ , accessed 22.04.2021.

10 Development assistance committee is an international forum of the largest providers of aid, including 30 members. See https://www.oecd.org/dac/development-assistance-committee/ , accessed 22.04.2021.

11 Framework for NGOs, Government of Bangladesh, see http://ngoab.portal.gov.bd/sites/default/files/files/ngoab.portal.gov.bd/notices/7158baa6_dac4_4f3b_8ff1_9ef680d32f71/Framework-for%20NGOs.pdf, accessed 10.10.2020

12 Foreign donations (voluntary activities) Regulation Act 2016, http://www.parliament.gov.bd/images/pdf/acts_of_10th_parliament/acts_of_12th_session/43.pdf, accessed 01.04.2021

13 CIVICUS monitor, available at https://monitor.civicus.org/country/bangladesh/, accessed 20.06.2021

14 2019 civil society organization sustainability index, see https://www.fhi360.org/sites/default/files/media/documents/csosi-asia-2019-report.pdf, accessed 15.06.2021

15 Local NGOs flagged UN practice of shrinking inclusiveness contrary to their own commitment. Press statement (CCNF). July 2020, see http://www.cxb-cso-ngo.org/wp-content/uploads/2020/07/CCNF-Statement-English_edited-30-July.pdf, accessed 17.05.2021
